# Novel PDI-NH/PDI-COOH Supramolecular Junction for Enhanced Visible-Light Photocatalytic Phenol Degradation

**DOI:** 10.3390/molecules29174196

**Published:** 2024-09-04

**Authors:** Yongzhang Xu, Xingrui Luo, Fulin Wang, Wentao Xiang, Chensheng Zhou, Weiya Huang, Kangqiang Lu, Shaoyu Li, Man Zhou, Kai Yang

**Affiliations:** 1 Jiangxi Provincial Key Laboratory of Functional Molecular Materials Chemistry, School of Chemistry and Chemical Engineering, Jiangxi University of Science and Technology, Ganzhou 341000, China; 2 Jiangxi Provincial Engineering Technology Research Center for Electronic Chemicals of Printed Circuit Boards, Ganzhou 341000, China; 3 School of Pharmaceutical Sciences, Gannan Medical University, Ganzhou 341000, China

**Keywords:** PDI-NH, PDI-COOH, heterojunction, S-scheme, phenol degradation

## Abstract

The development of efficient and environmentally friendly photocatalysts is crucial for addressing global energy and environmental challenges. Perylene diimide, an organic supramolecular material, holds great potential for applications in mineralized phenol. In this study, through the integration of different mass ratios of unmodified perylenimide (PDI-NH) into the self-assembly of amino acid-substituted perylenimide (PDI-COOH), a novel supramolecular organic heterojunction (PDICOOH/PDINH) was fabricated. The ensuing investigation focuses on its visible-light mineralized phenol properties. The results show that the optimal performance is observed with a composite mass fraction of 10%, leading to complete mineralization of 5 mg/L phenol within 5 h. The reaction exhibits one-stage kinetics with rate constants 13.80 and 1.30 times higher than those of PDI-NH and PDI-COOH, respectively. SEM and TEM reveal a heterogeneous interface between PDI-NH and PDI-COOH. Photoelectrochemical and Kelvin probe characterization confirm the generation of a built-in electric field at the interface, which is 1.73 times stronger than that of PDI-COOH. The introduction of PDI-NH promotes π-π stacking of PDI-COOH, while the built-in electric field facilitates efficient charge transfer at the interface, thereby enhancing phenol decomposition. The finding demonstrates that supramolecular heterojunctions have great potential as highly effective photocatalysts for environmental remediation applications.

## 1. Introduction

Water has always played a vital role in various fields such as industry and agriculture. However, a large number of pollutants (e.g., phenol, nitrobenzene and rhodamine B) have been discharged into the water with the rapid development of industry, resulting in increasingly serious water pollution [1,2]. To overcome this issue, advanced oxidation, physical adsorption, photocatalysis, and other approaches are employed to degrade the pollutants in the water [3,4]. Photocatalysis based on semiconductors for degrading pollutants is of high interest because of its environmental friendliness and economic sustainability. At present, photocatalysts such as Bi-based materials have been widely investigated in the degradation of organic pollutants [5,6,7,8]. However, these catalysts exhibited poor recoverability and photocatalytic performance. On the other hand, some photocatalysts with precious metals such as AgI have excellent photocatalytic performance due to their narrow band gap, but they are expensive and have poor durability, a fact that drastically limits their widespread application [9,10]. Therefore, it is crucial to find an affordable photocatalyst with outstanding degradation properties. 

Perylene diimide (PDI) is an n-type organic semiconductor material, while PDI-NH is the derivative of PDI, i.e., a supramolecule with the unique π-π conjugation plane formed through a polybenzene ring [11,12,13]. PDI-NH has excellent stability and a large light absorption range, although it only contains non-metallic elements of C, H, O, and N. Furthermore, due to their lower cost and eco-friendliness, PDI-NH semiconductors have become a promising photocatalyst [14,15,16]. Unfortunately, the photocarrier migration of PDI-NH is unstable due to the poor molecular force. Hence, the photocatalytic performance of PDI-NH still requires further improvement. In recent decades, it has been found that constructing a heterojunction of PDI-NH with other semiconductors (e.g., porphyrin, C_3_N_4_, polyaniline) could effectively enhance the photocatalytic performance of PDI-NH, which provides possibilities for the modification of PDI-NH [17,18,19,20].

PDI-COOH is another derivative material of PDI. PDI’s hydrophobicity can be enhanced by the added carboxyl group, leading to the tight binding of PDI-COOH with the other semiconductors [21]. And the migration efficiency of PDI-COOH has been improved, causing PDI-COOH to attract much attention [22]. PDI-COOH is frequently combined with other semiconductors to form heterojunctions [23], such as the PDI-COOH/iron phthalocyanine heterojunction. This construction enhances the migration efficiency of photocarriers and modulates the photocatalytic activity of PDI-COOH. Given these benefits, establishing the PDI-NH/PDI-COOH S-Scheme heterojunction holds significant importance.

The primary focus of this research is to introduce PDI-NH into the self-assembly process of multilayer structures alongside PDI-COOH, aiming to create a PDI-NH/PDI-COOH organic material featuring an S-Scheme heterojunction, and to assess its photocatalytic efficacy in phenol degradation. The synthesis of PDI-NH/PDI-COOH and the subsequent mineralization reaction during the photocatalytic process were scrutinized through various characterization methods to identify the underlying reasons for the enhancement in photocatalytic performance. Finally, a plausible mechanism was postulated based on the outcomes of the aforementioned analyses. 

## 2. Results and Discussion

### 2.1. Morphology and Structure Analysis

**Figure 1:**
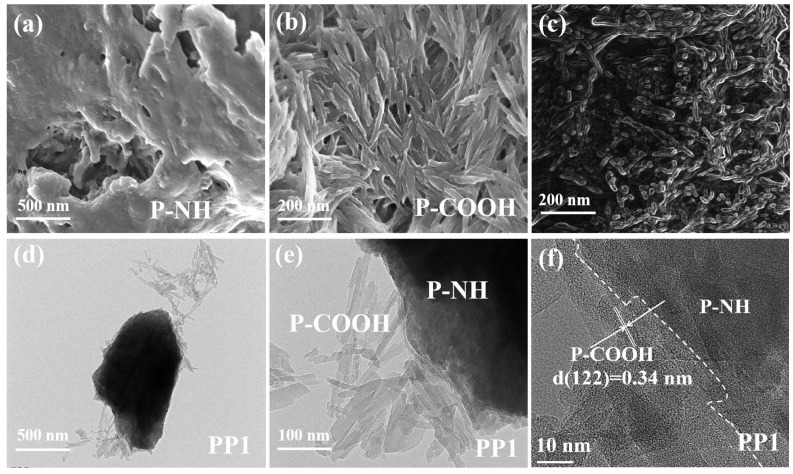
SEM diagram of PP series samples: (**a**) P-NH; (**b**) P-COOH; (**c**) PP-1; (**d**–**f**) TEM and HRTEM images of PP-1.

**Figure 2:**
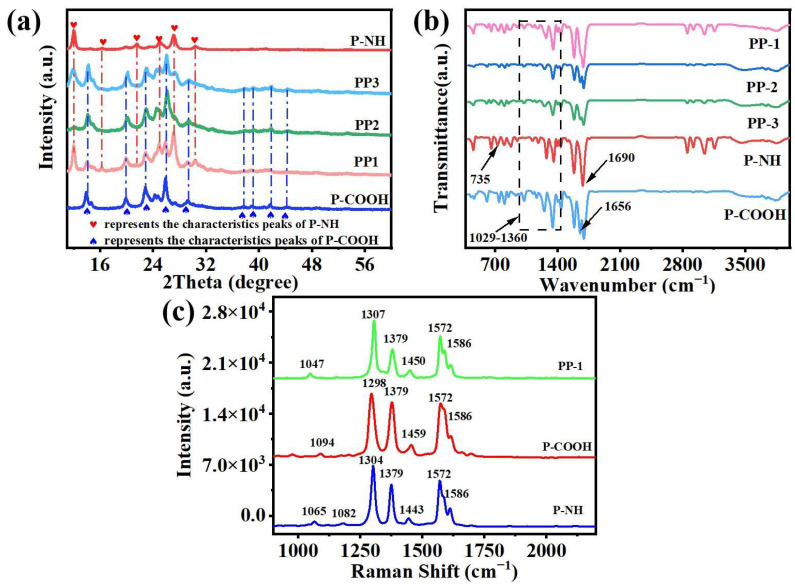
(**a**) XRD patterns, (**b**) Fourier transform infrared spectra, and (**c**) Raman spectra of PP series samples.

The morphology of the materials was examined using SEM. Figure 1a illustrates the irregular lumpy morphology of P-NH, suggesting its ability to offer ample surface area for the growth of other substances, aligning with the intended material structure. Conversely, P-COOH exhibits a nanorod morphology, as depicted in Figure 1b. On the heterojunction of PP (Figure 1c), a higher density of nanorods is evident on the bulk structure’s surface, indicating the formation of the PP photocatalyst through the encapsulated growth of P-COOH on P-NH’s surface. This configuration significantly increases the contact area with organic pollutants compared to the individual samples [15]. TEM analysis was conducted to investigate the microstructure of the sample, showing a distribution of the PP heterojunction in alignment with SEM findings (Figure 1d,e). The presence of P-COOH on the surface of P-NH is confirmed, leading to the formation of a conducive contact interface, as highlighted in the magnified images in Figure 1f. The structures of the prepared photocatalysts were characterized by XRD. As is shown in Figure 2a, the characteristic peaks of P-NH are located at 2θ = 12°, 16°, 21°, 25°, 27° and 30°, while the peaks of P-COOH are located at 14°, 20°, 22°, 25°, 29°, 37°, 38°, 42° and 44° [24]. It is clear that they have similar characteristic peaks due to the fact that they have a similar polybenzene ring structure. In addition, P-COOH exhibits the unique “double peak” feature in the range of 20° to 26° due to self-assembly and stacking to form a multilayered structure [25]. In Figure 2a, the characteristic peaks of P-COOH and P-NH are evident in the PP series heterojunction. As the P-NH content in the heterojunction increases, the intensity of the P-NH peak gradually rises, aligning with the content change pattern. The self-assembly degree of P-COOH increases with the peak value of P-NH, resulting in a higher presence of a π-π stacking structure and an accelerated photogenerated electron migration rate. The specific surface area of the sample was determined through N_2_ adsorption–desorption analysis, with the results presented in the accompanying Figure S1. The specific surface areas of P-COOH, P-NH, and PP-1 were measured at 1.5 m^2^/g, 20.4 m^2^/g, and 14.0 m^2^/g, respectively. PP-1 exhibits a larger specific surface area compared to P-COOH, attributed to the incorporation of P-NH with a higher specific surface area. The enhanced specific surface area of PP-1 facilitates an increased availability of active sites, thereby promoting the adsorption and catalytic degradation of phenol. 

FT-IR spectra were employed for the characterization of distinct functional groups present in the samples. In Figure 2b, the benzene ring’s C-H bond peak is at 735 cm^−1^, while its C-C and C=C bonds show peaks at 1000–1300 cm^−1^ and 1656 cm^−1^, respectively. Additionally, the PDI’s C=O bond displays a diffraction peak at 1688 cm^−1^ [26,27]. The presence of the benzene ring and the original C=O bond of perylene diimide in both P-NH and P-COOH samples results in the presence of all four peaks in their spectra. However, a notable distinction is observed in P-NH, where a continuous range of peaks between 3000 and 3300 cm^−1^ corresponds to the characteristic N-H bond in peryleneimide, a feature not present in P-COOH [28]. The persistent peak is similarly detected in the composite samples, with the sharpness of these peaks intensifying as the P-NH content increases. The utilization of Raman spectra allows for further characterization of both the valence bond and the expansion of functional groups. In Figure 2c, peaks ranging from 1500 to 1600 cm^−1^ are attributed to the stretching vibrations of the C–C, C=C, and C=O bonds of perylene imide, while the peak at 1300 cm^−1^ is associated with the stretching vibrations of the C–H bond [29]. The variation in the peak value at 1586 cm^−1^ indicates differing intermolecular π-π packing strengths, with P-COOH exhibiting the strongest packing strength and P-NH the weakest. The positional and intensity changes of the peaks of P-NH, P-COOH, and PP-1 suggest that the introduction of P-NH modulates the accumulation of P-COOH, establishes a suitable delocalized π–electron conjugation system for the S-Scheme heterojunction, and thereby enhances the efficiency of photoinduced carrier separation [30]. 

**Figure 3:**
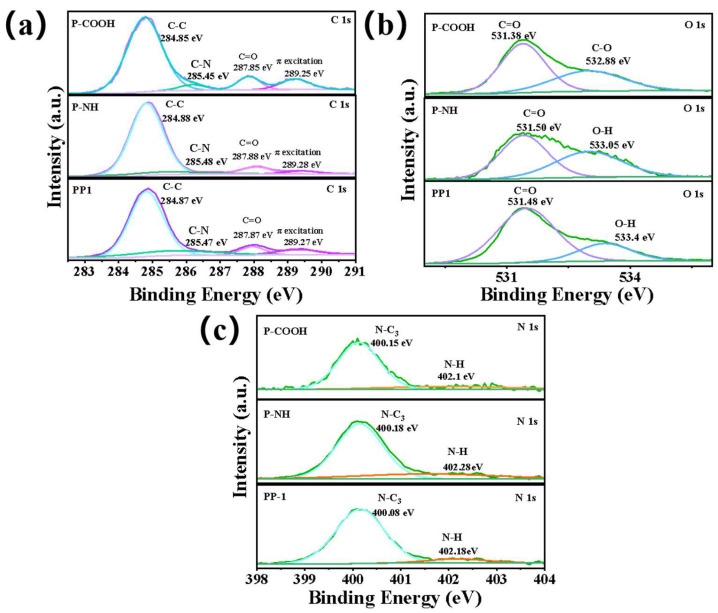
XPS spectra of PP series samples: (**a**) C 1s; (**b**) O 1s; (**c**) N 1s.

XPS was utilized to examine the valence states of P-NH and P-COOH on the PP photocatalyst. Figure 3 illustrates the presence of carbon (C), nitrogen (N), and oxygen (O) in PP1, P-COOH, and P-NH. In the PP1 spectra, peaks at binding energies of 284.85, 285.45, and 287.85 eV correspond to the C–C, C–N, and C=O bonds in P-COOH, respectively (Figure 3a). Notably, there is no significant shift in these bonds’ binding energies, indicating the structural stability of P-COOH in PP1. Furthermore, the π–electron excitation peak at 289.25 eV suggests a π-π stacking structure in P-COOH [23]. In comparison to P-NH and PP-1, the π–electron excitation peak strength is notably highest in P-COOH, aligning with XRD findings. The spectrum of P-COOH in Figure 3b displays characteristic peaks of binding energy at 531.38 and 532.88 eV, attributed to the C=O and C–O bonds at the carboxyl-terminal position. Additionally, in P-NH, the binding energies of the characteristic peaks for C=O and O–H are 531.50 eV and 533.05 eV, respectively [18]. The comparison of the binding energies of O–H in PP1 and P-NH in Figure 3b reveals a higher binding energy for O–H in PP1 than in P-NH, indicating a clear interaction between P-NH and P-COOH, with electron flow from P-NH to P-COOH. In Figure 3c, the peaks observed at 400.15 and 402.28 eV on the PP series samples correspond to N-C3 and N–H bonds. The binding energies of N–C and N–H in P-NH are 400.18 eV and 402.28 eV, respectively. In PP-1, the binding energies of N-C3 and N–H are 400.08 eV and 402.18 eV, respectively. Comparatively, the binding energy of N-C3 in PP-1 is lower than that in P-COOH, indicating partial electron transfer from P-NH to P-COOH. In conclusion, XPS analysis confirms the presence of C, N, and O elements in PP-1 and reveals the electron flow from P-NH to P-COOH. The introduction of P-NH implies the creation of efficient charge transmission within PP. Furthermore, the band structure analysis of P-NH and P-COOH indicates the formation of an S-Scheme heterojunction between them. 

### 2.2. Photocatalytic Phenol Degradation 

**Figure 4:**
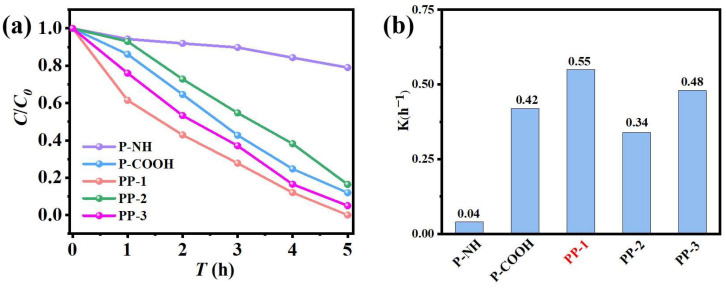
(**a**) Degradation efficiency of phenol over different photocatalysts, (**b**) pseudo-first-order kinetic of phenol.

To evaluate the photocatalytic performance of the catalyst, a solution containing 5 mg/L of phenol was subjected to photocatalytic mineralization under 400 W metal halide light (300~800 nm) for 5 h. The degradation efficiency of P-NH towards phenol was found to be 21% after 5 h of light exposure, as depicted in Figure 4a. Upon combining the two bare substances in varying ratios, the degradation efficiencies for phenol were determined to be 100% for PP-1, 84% for PP-2, and 89% for PP-3. Upon examining the total organic carbon (TOC) levels in the phenol solution following the reaction over PP1, it is observed that the TOC value decreases progressively as the illumination time extends, as detailed in Table S1 below. Subsequently, after a duration of five hours, the TOC concentration reached 0.002 mg/L, indicating the effective mineralization of phenol. This disparity is attributed to the poor hydrophilicity of P-NH, leading to limited interaction with the phenol solution. Conversely, P-COOH and the PP series catalysts, featuring terminal carboxyl groups and improved hydrophilicity, exhibit enhanced phenol degradation efficiencies. To elucidate the driving force governing the catalyst reaction mechanism, the degradation rate constant was calculated based on the pseudo-first-order kinetic model. The sequence of degradation rate constants, as illustrated in Figure 4b, is as follows: PP-1 > PP-3 > P-COOH > PP-2 > P-NH. Of significance, PP-1 displays the highest *k* value of 0.55 among the samples, indicating both superior photocatalytic performance and a more robust reaction driving force [31]. Additionally, the photocatalytic performance of PP-3 outperforms that of PP-2, indicating an enhancement in the heterojunction’s efficiency with an increase in the P-NH content on the composite. This improvement is likely due to the higher concentration of P-NH, which aids in providing a more extensive electron transport platform and expediting carrier transport [32]. 

### 2.3. Active Species and Stability

**Figure 5:**
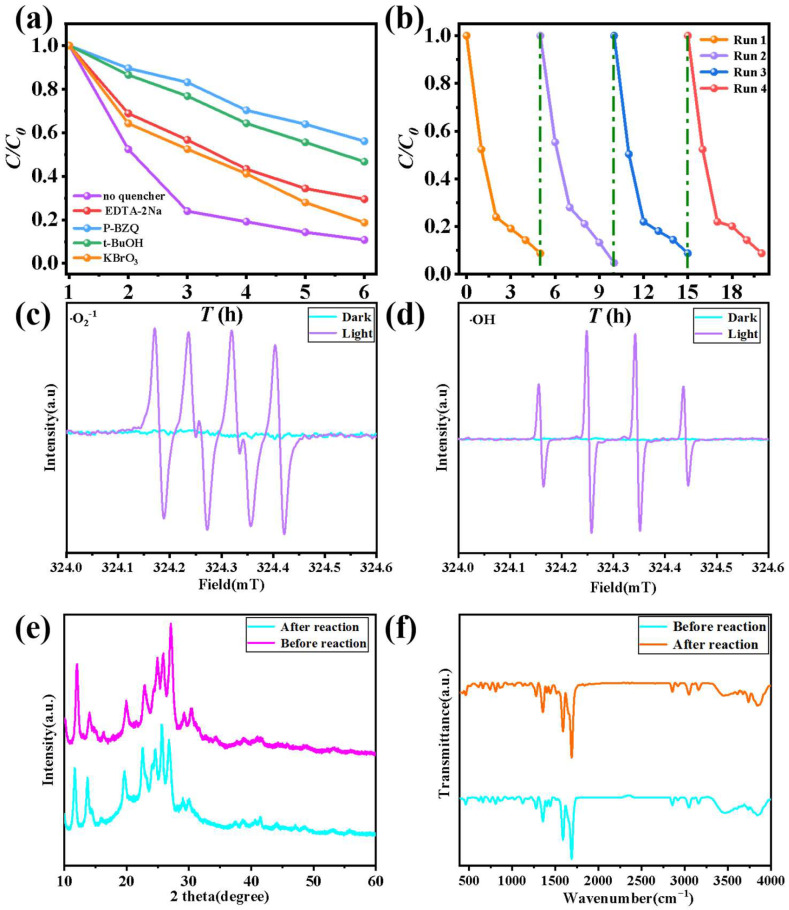
Trapping agent experiments (**a**) and cycle experiments (**b**) of PP-1 sample; EPR signals of DMPO-·O_2_^−^ (**c**) and DMPO-·OH (**d**) adducts (irradiation for 10 min) generated by PP1. XRD (**e**) and FT-IR (**f**) results of PP1 samples before and after reaction.

To investigate the primary active species involved in the photocatalytic mineralization of phenol using the PP-1 photocatalyst, a reactive oxygen species (ROS) experiment was conducted. During the degradation of a 5 mg/L phenol solution, 1 mmol of tert-butanol (t-BuOH), potassium bromide (KBrO_3_), EDTA-2Na, and p-benzoquinone (P-BZQ) were introduced as scavengers for hydroxyl radicals, photoexcited electrons and holes, and superoxide radicals, respectively. The results depicted in Figure 5a reveal that upon the addition of t-BuOH and P-BZQ, the phenol degradation efficiencies of PP-1 decrease from 90.3% to 53.4% and 44.9%, respectively. Conversely, the introduction of KBrO_3_ leads to a slight reduction in degradation efficiency from 90.3% to 81.3%. These findings suggest that superoxide radicals (·O_2_^−^) and hydroxyl radicals (·OH) are pivotal in the mineralization of phenol by the PP photocatalyst, while electrons and holes play a secondary role. Four cycling experiments were conducted to assess the stability of the PP-1 catalyst. The results, as depicted in Figure 5b, indicate that PP-1 maintains stable photocatalytic performance, achieving a degradation efficiency exceeding 95.0% after four cycles. The EPR test provides evidence for the existence of ·O_2_^−^ and ·OH adducts (irradiation for 10 min) generated by PP1, as illustrated in the following Figure 5c,d. XRD patterns in Figure 5e and FT-IR spectra in Figure 5f results were used to analyze the structural changes of the optimal PP-1 photocatalyst before and after the catalytic reactions, revealing no significant changes in the PP-1 structure. The morphology from SEM in Figure S2 of PP1 also demonstrates that it is largely unaffected. These findings demonstrate the exceptional photocatalytic activity and structural robustness of the PP-1 photocatalyst.

### 2.4. Optical Absorption

**Figure 6:**
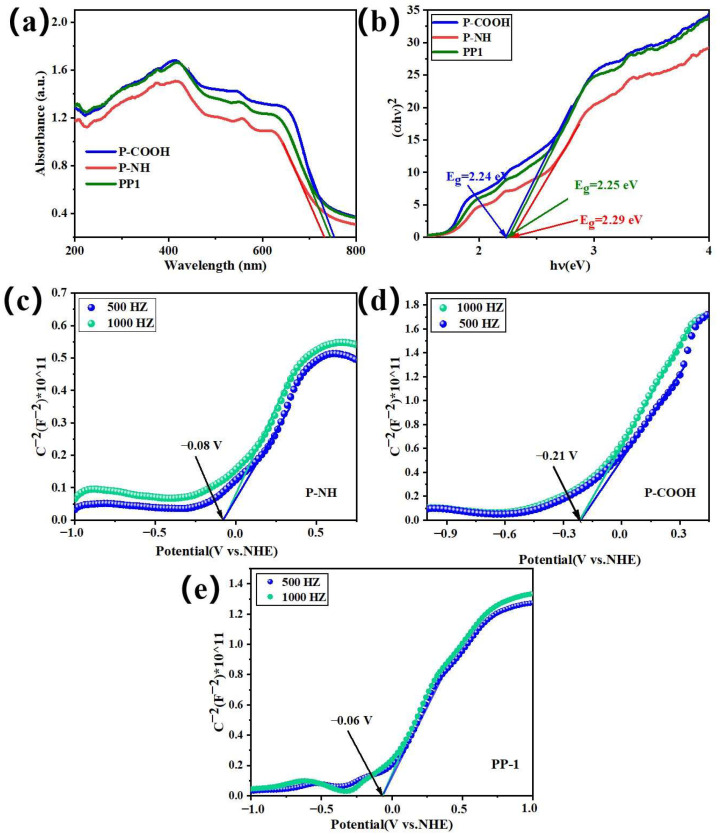
UV-Vis DRS spectra (**a**) and (*αhν*)*^2^-hν* diagrams (**b**) of PP series samples; Mott–Schottky curves of P-NH, PP-1and P-COOH (**c**–**e**).

Through UV-Vis DRS spectra, the optical characteristics of photocatalysts were assessed. The proximity of the band gap absorption peaks of P-NH, P-COOH, and PP-1 to 750 nm (Figure 6a) indicates the extensive light absorption capacity of PP-1. The band gap of the semiconductor can be obtained by the Tauc equation of ${\left(\alpha hv\right)}^{1/n}=A(hv-E_{g})$. As P-COOH is a direct semiconductor, the value of *n* in this scenario is 2 [33]. Figure 6b illustrates the computed outcomes, demonstrating that P-COOH possesses the smallest band gap of 1.72 eV, whereas P-NH exhibits a wider band gap of 2.31 eV. The merging of P-NH and P-COOH results in a band gap reduction to 2.18 eV in PP-1, suggesting that their recombination involves an interfacial interaction rather than a simple stacking arrangement. This interaction facilitates the creation of a heterojunction, thereby influencing the band gap.

The energy level structure of the semiconductor was determined through the measurement of the M-S curve of the sample. Analysis of Figure 6c–e reveals that all photocatalysts exhibit a positive slope in their M-S curves, indicating their classification as n-type semiconductors. The flat-band potential (*E_fb_*) of the semiconductor is identified at the intersection of the curve and the *X*-axis [34]. The *E_fb_* values of P-NH, P-COOH and PP-1 is −0.08, −0.21, and −0.06 eV, respectively. Since the conduction band potential (*E_CB_*) of n-type semiconductors can be determined by shifting the flat-band potential negatively by 0.2 eV, the *E_CB_* of P-NH, P-COOH, and PP-1 is −0.28, −0.41, and −0.26 eV, respectively. According to the formula *E_VB_ = E_g_ + E_CB_*, the valence band potential (*E_VB_*) of P-NH, P-COOH, and PP-1 can be obtained as 2.29, 2.24, and 2.25 eV, respectively (Table 1). This research highlights an energy level crossover between the two components, hinting at the potential development of an S-Scheme heterojunction. 

### 2.5. Charge Transfer Properties

**Figure 7:**
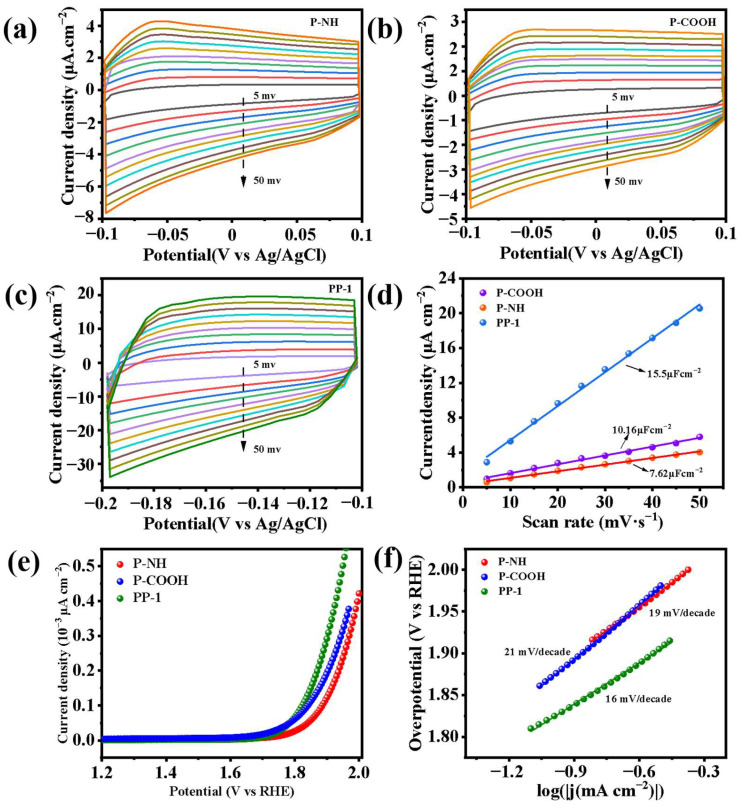
**CV curves** and LSV curves of PP series samples: (**a**) P-NH; (**b**) P-COOH; (**c**) PP-1; (**d**) current density–scan rate plots; (**e**) LSV polarization curves; (**f**) Tafel slope plots.

Utilizing cyclic voltammetry (CV) curves enables the quantification of the active surface area of the heterojunction catalyst and the evaluation of electron transport efficiency at the interface [35]. Figure 7a–c illustrate an increase in the capacitance current value with higher scan rates, indicating a positive association between capacitance current and the catalyst’s active surface area. To compare changes in specific surface areas of composite and pure samples more effectively, the relationship between current density and scan rate can be analyzed. The slope of the resultant line in the figure can then be used to determine the double-layer capacitance (*C_dl_*). Notably, in Figure 7d, the *C_dl_* value of PP-1 (15.5 μF·cm^−2^) surpasses that of P-NH (7.62 μF·cm^−2^) and P-COOH (10.16 μF·cm^−2^). This disparity suggests that the composite enhances the active surface area, thereby providing additional active sites to catalyze reactions and improve photocatalytic efficiency.

The determination of the oxidizing ability of the PP catalysts is achievable through the combination of LSV curves with Tafel slopes [36]. From Figure 7e, it can be concluded that PP-1 has the smallest overpotential, while P-NH has the largest overpotential, indicating that PP-1 needs to cross the smallest energy barrier and has the best oxidative property at the same current density. The kinetic curve of oxidation performance, i.e., the Tafel slope plot, was further obtained from the LSV curve. In Figure 7f, the Tafel slope of PP-1 is 16 mV/decade, while the Tafel slopes of single-component P-NH and P-COOH are 19 mV/decade and 21 mV/decade, respectively. Catalysts exhibiting smaller Tafel slopes are indicative of higher charge transfer efficiencies and lower resistance during charge transfer. As a result, PP-1 displays superior charge transfer efficiency and improved oxidizability.

**Figure 8:**
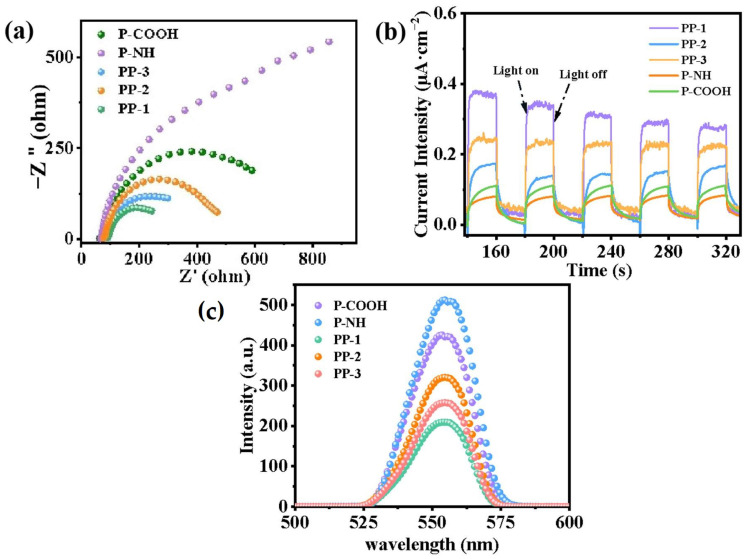
Photochemical properties of PP series samples: (**a**) transient photocurrent; (**b**) electrochemical impedance spectra; (**c**) steady-state fluorescence spectra, *λ_ex_* = 340 nm.

Photoelectrochemical experiments were conducted to investigate the photocharge transfer in the PP series catalysts. The electrochemical impedance curve illustrates the impedance levels of photogenerated electrons as they traverse the sample. Figure 8a displays the electrochemical impedance spectrum of the catalyst in a solution containing potassium ferricyanide and potassium ferricyanide. The findings reveal that the curvature radius of the samples follows the order P-NH > P-COOH > PP-2 > PP-3 > PP-1. A smaller curvature radius in the impedance spectrum corresponds to lower resistance during charge transfer and enhanced photogenerated carrier migration capability. Consequently, PP-1 exhibits the highest rate of photogenerated carrier migration. In Figure 8b, a comparison of the current magnitude of the PP series catalyst under interval illumination in a 0.1 M Na_2_SO_4_ solution is depicted [37]. The photocurrent in all PP samples surpasses that of P-COOH and P-NH samples, with PP-1 exhibiting the highest intensity and P-NH the lowest. This trend suggests that higher P-NH content enhances the photoelectric response and carrier separation efficiency, leading to increased generation of photoelectrons.

Utilizing the steady-state fluorescence spectra allows for the assessment of the carriers’ complexation efficiency in the catalyst [38]. Figure 8c demonstrates that P-NH exhibits the highest excitation intensity at a wavelength of 340 nm upon complexation with P-COOH. In contrast, the excitation intensity decreases significantly after complexation with P-COOH, with PP-1 displaying the lowest intensity. This suggests that the formed P-NH/P-COOH composite exhibits reduced photoexcited carrier complexation in the heterojunction, which is conducive to phenol mineralization. Overall, the photoelectrochemical properties of the PP catalysts surpass those of the individual components. Notably, PP-1 displays the highest photocurrent, lowest impedance, and slowest carrier recombination rate. These findings indicate that PP-1 can generate more photogenerated electrons and holes, and possesses enhanced charge mobility and utilization efficiency during photocatalytic reactions.

### 2.6. Contact Angle

**Figure 9:**
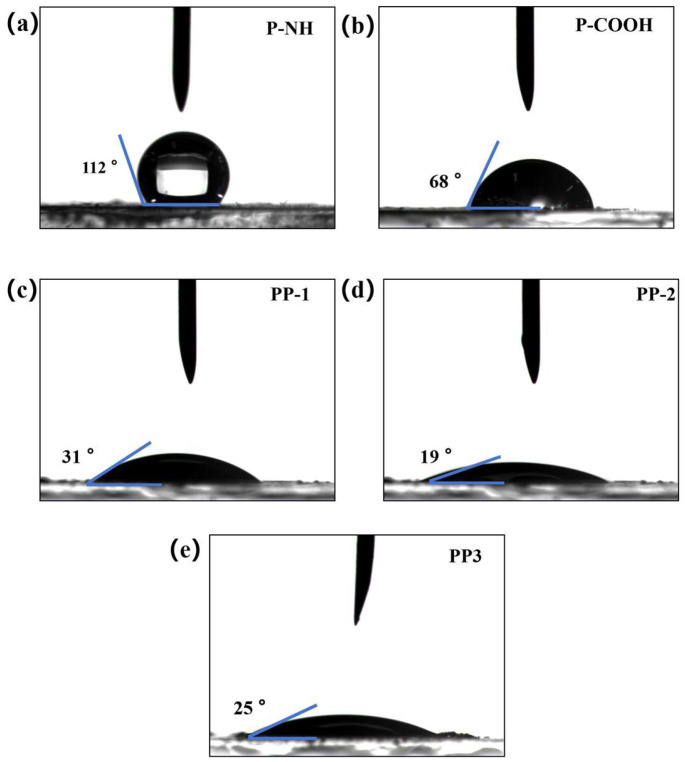
Schematic diagrams of contact angles in the phenol solution of P-NH (**a**); P-COOH (**b**); PP-1 (**c**); PP-2 (**d**); and PP-3 (**e**).

The contact angle test indicates the surface tension of the catalyst when in contact with the reaction liquid. A smaller contact angle value signifies a higher affinity between the solution and the catalyst, facilitating better wrapping of the catalyst surface by the liquid [39]. As shown in Figure 9, the contact angle between P-NH and phenol solution is 112° and the affinity is poor, which means that the active site of P-NH cannot be fully utilized, resulting in poor photocatalytic activity. The contact angle of P-COOH is 68°, which is due to the introduction of a hydrophilic carboxyl group at the terminal position of P-COOH to produce a hydrogen bond, thus promoting the wetting of phenol solution to the catalyst. The PP-1, PP-2, and PP-3 catalysts exhibit smaller contact angles of 31°, 19°, and 25°, significantly lower than those of P-NH and P-COOH. This suggests a strong affinity of PP-1 for phenol, enhancing the adsorption of phenol molecules and increasing the availability of active sites, thereby improving the photocatalytic performance.

### 2.7. Built-in Electric Field and Mechanism

**Figure 10:**
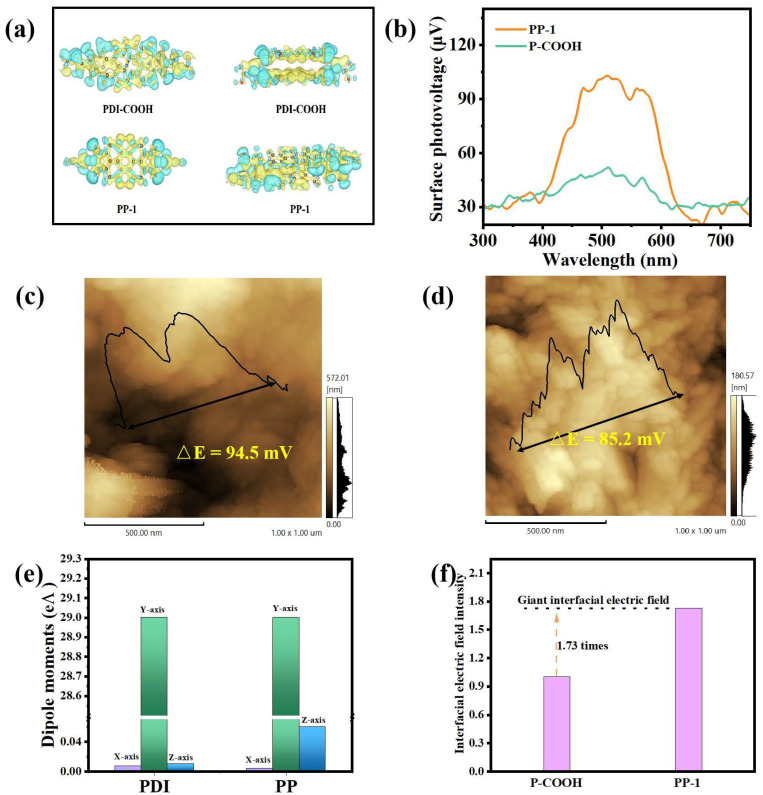
(**a**) Differential charge densities of P-COOH and PP-1 (yellow represents electrons aggregation state, blue represents the consumption of electrons); (**b**) surface optical voltages; surface potential differences of PP-1 (**c**) and -COOH (**d**); (**e**) molecular dipole moments (Purple represents the dipole moment in the x-axis direction, green represents the y-axis direction and blue represents the z-axis direction.); (**f**) intensities of built-in electric field.

The feasibility of the envisioned charge separation system can be evaluated through a systematic investigation of the structure and interfacial charge of a heterojunction using density functional theory and KPFM [31,40]. Figure 10a displays the charge density contrast between P-COOH and PP-1 in different orientations, enabling the assessment of atomic bonding in the optimized structural model. Upon comparing P-COOH and PP-1, it is evident that electron redistribution occurs following the recombination of P-NH and P-COOH. The yellow region signifies electron aggregation, and the consequent unequal distribution of electrons among atoms induces changes in dipole moments. This modification is a crucial prerequisite for the generation of a built-in electric field at the interface [41]. Figure 10b illustrates a comparison of the surface photovoltage among the catalyst samples. The data indicate that PP-1 exhibits a higher surface photovoltage, suggesting an efficient electron transfer process within the heterojunctions, thereby facilitating the separation of photoinduced carriers. In order to further investigate the built-in electric field strength situation, the surface potential of the sample was probed using KPFM [42]. The surface potential of PP-1, illustrated in Figure 10c,d, is notably stronger at 94.5 mV compared to P-COOH, which registers at 85.2 mV.

The electron transfer at the interface induces a variation in the dipole moment, enabling the detection of alterations in the internal electric field. Figure 10e illustrates that, in comparison to P-COOH, the dipole moment of PP-1 exhibits minimal changes along the *Y*-axis but displays discrepancies along the X and Z axes. This phenomenon arises from the shared perylene imide core structure of P-COOH and PP-1, which constitutes a conjugated structure formed through molecular accumulation. Changes in the dipole moment along the X and Z axes signify that the incorporation of P-NH introduces the intrinsic electric field, thereby facilitating enhanced charge separation. A correlation was observed among surface potential, photocurrent density, and the intensity of the built-in electric field. The values of the electric field were determined by combining current charge density and surface photovoltage, as depicted in Figure 10f. It is revealed that the built-in electric field intensity of PP-1 exceeds that of P-COOH by a factor of 1.73. This suggests that the electric field within the PP-1 heterojunction interface is more conducive to the separation and directional movement of carriers, thereby enhancing the efficiency of utilizing photogenerated electrons and holes in PP-1. Consequently, the mineralization process of phenol was significantly accelerated.

The photodegradation mechanism of phenol by PDINH-PDICOOH was investigated by analyzing the band structures of P-NH and P-COOH, along with experimental data on photoactivity. The study revealed an “energy level crossing” phenomenon between P-NH and P-COOH, leading to two hypotheses: one suggesting an organic type II heterojunction and the other proposing an organic S-Scheme heterojunction. In the case of an organic type II heterojunction, photoelectrons from P-COOH’s conduction band could transfer to P-NH’s conduction band, while holes from P-NH’s valence band could transfer to P-COOH’s valence band. Capture experiments confirmed that the decomposition of phenol by the PP catalyst is primarily driven by superoxide and hydroxyl radicals. Furthermore, it is observed that only P-COOH’s conduction band meets the necessary potential for the superoxide radical, while P-NH does not. This leads to the retention of P-COOH’s conduction band and P-NH’s valence band in the heterojunction, indicating that a type II heterojunction alone is insufficient to explain the photodegradation mechanism of the PP catalyst induced by phenol.

**Figure 11:**
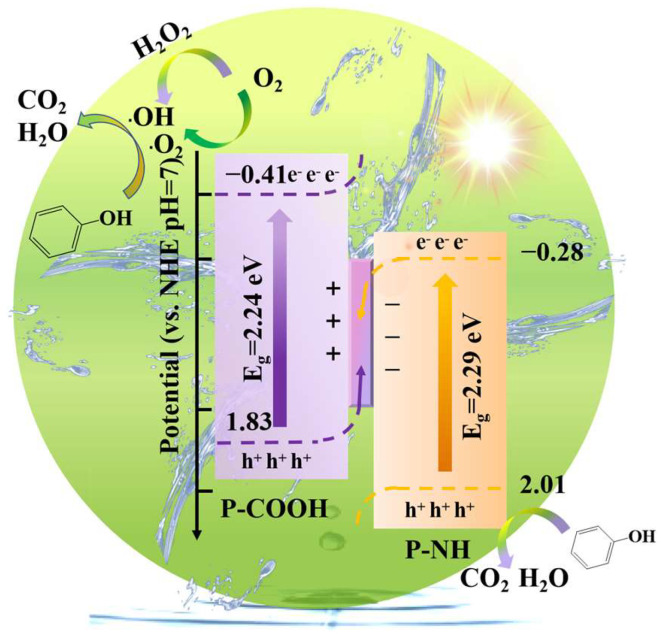
Possible mechanism of photocatalytic phenol mineralization with PDI-NH/PDI-COOH supramolecular junction.

The mechanism diagram in Figure 11 illustrates that in the S-Scheme heterojunction, holes from the P-COOH valence band can combine with photoelectrons from the P-NH conduction band. This aligns with the hypothesis that the trap experiment captures the P-COOH conduction band and P-NH valence band. Photogenerated electrons in the P-COOH conduction band may react with O_2_ and H_2_O to form superoxide radicals (–O_2_^−^) and hydroxyl radicals (–OH), leading to the decomposition of phenol into smaller molecular intermediates and eventual mineralization into CO_2_ and H_2_O. Additionally, the P-NH valence band retains photogenerated holes that actively facilitate the phenol degradation process. Consequently, the S-Scheme junction provides valuable insights into the photodegradation mechanism of phenol on PP catalyst under visible-light exposure.

## 3. Experimental Section

### 3.1. Materials

Ethanol (C_2_H_6_O, AR) and β-alanine (C_3_H_7_NO_2_, AR) were purchased from Shanghai Aladdin Biochemical Science and Technology Co. (Shanghai, China) 3,4,9,10-Perylenetetracarboxylic acid dianhydride (C_24_H_8_O_6_, AR) and triethylamine (C_6_H_15_N, AR) were purchased from Shanghai McLean Biochemical Technology Co. (Shanghai, China). Imidazole (C_3_H_4_N_2_, AR). Concentrated hydrochloric acid (HCl, AR) Imidazole (C_3_H_4_N_2_, AR), concentrated hydrochloric acid (HCl, AR) and phenol (C_6_H_7_O, AR) were purchased from Xilong Technology Co. (Shantou, China). 

### 3.2. Preparation of Polyxylene-3,4,9,10-Tetracarboxylic Dianhydride

**Scheme 1:**
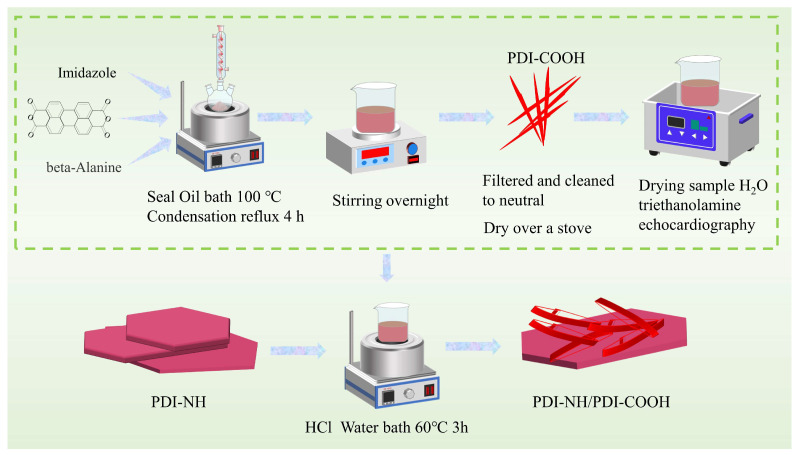
Synthesis of PDI-NH/PDI-COOH.

The synthesis steps are shown in Scheme 1. PDI-COOH was synthesized as follows [23]: Firstly, 1.376 g (3.5 mmol) of polyxylene-3,4,9,10-tetracarboxylic dianhydride, 2.5 g (28 mmol) of β-aminopropionic acid, and 18 g (0.265 mol) of imidazole were placed into a 100 mL triple-necked flask, stirred, and passed through helium for 15 min. The product was then condensed and refluxed at 100 °C in an oil bath for 4 h. Subsequently, the product was transferred to a beaker before the imidazole solidified, the residual product was washed with 100 mL of anhydrous ethanol and 300 mL of 2 M hydrochloric acid solution into the beaker, and the whole liquid was cooled naturally and stirred for 8 h. Finally, the product was filtered under vacuum, washed with deionized water to pH = 7 and dried at 60 °C for 12 h. 

### 3.3. Synthesis of PDI-NH/PDI-COOH

The preparation method of PDI-NH/PDI-COOH is as follows: Firstly, 0.5 g of dried dark red fragmentary PDI-COOH was placed into a 200 mL glass beaker, followed by the addition of 100 mL deionized water and subsequent dissolution through ultrasonic treatment. Subsequently, 800 μL triethylamine was introduced to the stirred liquid, and the mixture was heated to 60 °C for a duration of 30 min. Then, varying amounts (0.05/0.1/0.2 g) of 9,10-tetracarboxylic dianhydride (PDI-NH) were added to the liquid and subjected to heating for another 30 min. During the preparation process, a solution of 4 M HCl was prepared and each sample received an addition of 35 mL thereof while stirring continuously for a period lasting three hours. Finally, the resulting mixture underwent centrifugation and washing with deionized water until neutralization was achieved. It was dried under vacuum conditions at a temperature of 60 °C. The prepared samples were designated as PDI-COOH (hereinafter referred to as P-COOH), with PDI-NH (being referred to as P-NH), PP-1, PP-2, PP-3. 

### 3.4. Evaluation of Photocatalytic Performances

The degradation of phenol solution at 5 mg/L under visible light was used as the standard to test the photocatalytic performance of the catalysts. The used equipment is a light reaction meter with a net container content of 50 mL. A total of 30 mg of catalyst was added to 50 mL phenol solution, and the mixture was ultrasonicated for 2 min to disperse the catalyst uniformly. The catalyst was placed in the reaction instrument and rotated and stirred for 1 h under dark conditions to achieve equilibrium of absorption and desorption. The phenol mineralization reaction was carried out with a 400 W full-spectrum gold halide lamp as the simulated light source (filtered ultraviolet light ≤ 420 nm). The illumination intensity was 148.36 mW·cm^−2^. The reaction temperature was maintained at 25 °C by circulating condensed water throughout the photocatalytic reaction. During the reaction process, the reaction liquid was always rotated, and 3 mL of reaction liquid was placed into the centrifugal tube every 1 h for backup. After the end of the photoreaction process, the liquid removed from the reaction process was centrifuged and the solid catalyst was thoroughly filtered by a 0.22 μm filter membrane. An examination of the variation in phenol solution concentration during the reaction process was conducted via high-performance liquid chromatography. The degradation efficiency in the reaction process was calculated by the formula, where *η* represents degradation efficiency, *C* represents the concentration of the phenol solution after the reaction, and *C*_0_ represents the concentration of the phenol solution before the reaction.

$$\eta =\frac{C-C_{0}}{C_{0}}\times 100\%$$

Utilizing the pseudo-first-order kinetic approach, simulations were conducted to ascertain the kinetic constant for phenol mineralization, enabling the determination of the catalyst’s degradation rate. The equation is as follows: $\ln(C/C_{0})=-kt$, where *k* is the degradation rate constant, and *t* is time. 

### 3.5. Characterization

X-ray diffraction (XRD) was utilized to analyze the crystal structure of the photocatalyst using a Bruker D8 X-ray powder diffractometer (Bruker AG, Ettlingen, Germany). The crystal structure of the samples was determined with the Bruker D8 X-ray powder diffractometer to ascertain the crystalline surface of the catalyst samples. The surface morphology and structural composition of the samples were investigated using the scanning electron microscope (SEM, Zeiss Sigma 300, Carl Zeiss AG, Oberkochen, Germany) and the high-resolution transmission electron microscope (HR-TEM, JEM 2200FS, JEOL Ltd., Tokyo, Japan). Ultraviolet–visible diffuse reflectance spectroscopy (UV-Vis DRS) of the catalysts was conducted using a UV-2600 spectrophotometer (Shimadzu Corporation, Kyoto, Japan) with BaSO_4_ as the reflectance standard sample to determine the band edge absorption of the catalysts. The chemical bond composition of the sample was analyzed using a Fourier transform infrared spectrometer (FT-IR) Nicolet 5700 (Nicolet Co., Ltd., Mountain, WI, USA). Total organic carbon (TOC) levels were measured by a total organic carbon analyzer (TA-1.0). Utilizing a Bruker A300 spectrometer (Bruker AG, Ettlingen, Germany), electron paramagnetic resonance (EPR) spectra were captured at 77 K. The composition and valence were determined with an X-ray photoelectron spectrometer [XPS, Thermo Scientific K-Alpha (Seymour Fisher Technologies, Inc., Waltham, MA, USA)]. Photocurrent (IT), electrochemical alternating current impedance (EIS), cyclic voltammetry curves (CV), polarization curves (LSV), and Mott–Schottky (MS) curve tests were conducted with a CHI660E workstation equipped with a 300 W xenon lamp as the light source. A homogeneous dispersion of the sample material was applied on the FTO glass surface as the working electrode, while a saturated Ag/AgCl reference electrode and a platinum counter electrode were used. The variation of the built-in electric field for different samples was measured with a Kelvin probe force microscope (KPFM), model Multimode 8. 

## 4. Conclusions

In this study, a novel organic composite of PDI-NH/PDI-COOH was synthesized using a self-assembly strategy, and its efficacy in degrading phenol solution was investigated. The PDI-COOH material uniformly grew on the surface of PDI-NH, forming a S-Scheme heterojunction interface. The concentration of PDI-NH was found to significantly impact the photocatalytic activity in phenol degradation. All S-Scheme heterojunctions demonstrated efficient photocatalytic activity in degrading phenol solution, with PP-1 displaying the highest degradation efficiency, surpassing that of the individual samples. The enhanced charge transfer efficiency in the PP-1 composite was attributed to its larger specific surface area and improved electron–hole pair separation. The establishment of new electron migration channels from PDI-NH to PDI-COOH effectively suppressed carrier recombination, generating more active species for the redox reaction and thereby boosting photocatalytic efficiency. This composite material shows promise as an efficient photocatalyst with potential applications in various photocatalytic reactions.

## Figures and Tables

**Table 1:** *E_g_*, *E_CB_*, and *E_VB_* of P-COOH, P-NH, and PP-1.

Semiconductors	*E_CB_* (vs. NHE)	*E_VB_* (vs. NHE)	*E_g_* (eV)
**P-COOH**	−0.41	+1.83	2.24
**P-NH**	−0.28	+2.01	2.29
**PP-1**	−0.26	+1.99	2.25

## Data Availability

Data are contained within the article and Supplementary Materials.

## Supplementary Materials

The following supporting information can be downloaded at: https://www.mdpi.com/article/10.3390/molecules29174196/s1.
