# Supplementary material for: Novel PDI-NH/PDI-COOH Supramolecular Junction for Enhanced Visible-Light Photocatalytic Phenol Degradation
Source: Molecules. 2024 Sep 4;29(17):4196. doi: 10.3390/molecules29174196 (PMC11397078; doi:10.3390/molecules29174196)
Supplement: Supplementary file 1 [file molecules-29-04196-s001.zip › molecules-3172422-supplementary.pdf]

Novel PDI-NH/PDI-COOH Supramolecular Junction for Enhanced Visible-Light Photocatalytic Phenol Degradation

Yongzhang Xu [1,†], Xingrui Luo [1,†], Fulin Wang [1], Wentao Xiang [1], Chensheng Zhou [1], Weiya Huang [1], Kangqiang Lu [1], Shaoyu Li [1,*], Man Zhou [2,*] and Kai Yang [1,*]

[1] Jiangxi Provincial Key Laboratory of Functional Molecular Materials Chemistry, School of Chemistry and Chemical Engineering, Jiangxi University of Science and Technology, Ganzhou 341000, China
[2] School of Pharmaceutical Sciences, Gannan Medical University, Ganzhou 341000, China
[*] Correspondence: 9120150135@jxust.edu.cn (S.L.); baiyuwawa-zhouman@163.com (M.Z.); yangkai@jxust.edu.cn (K.Y.)
[†] These authors contributed equally to this work.

Table S1. Total organic carbon (TOC) levels in the photocatalytic degradation of phenol solution of 5 mg/L over PP1.

Time (h)	0	1	2	3	4	5
TOC (mg/L)	5.13	4.11	3.03	1.92	0.94	0.002

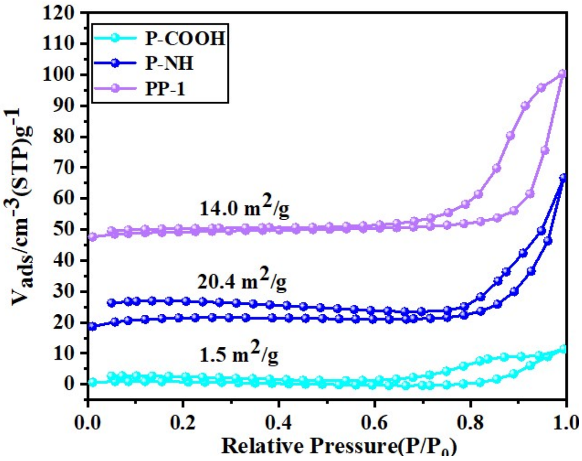

Figure S1. N2 adsorption-desorption diagrams of P-NH, P-COOH and PP-1.

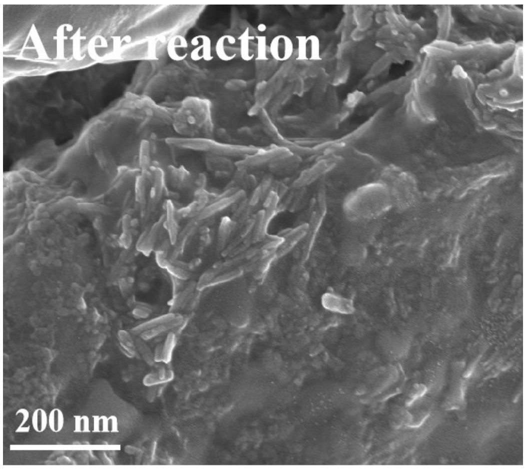

Figure S2. SEM image of PP-1 for post-reaction.
